# Supplementary material for: Spatiotemporal transcriptomic profiling and modeling of mouse brain at single-cell resolution reveals cell proximity effects of aging and rejuvenation
Source: bioRxiv. 2024 Jul 19:2024.07.16.603809. Preprint. [Version 1] doi: 10.1101/2024.07.16.603809 (PMC11275735; doi:10.1101/2024.07.16.603809)
Supplement: Supplement 17 [file NIHPP2024.07.16.603809v1-supplement-17.pdf]

bioRxiv preprint doi: https://doi.org/10.1101/2024.07.16.603809; this version posted July 19, 2024. The copyright holder for this preprint (which was not certified by peer review) is the author/funder, who has granted bioRxiv a license to display the preprint in perpetuity. It is made available under aCC-BY 4.0 International license.

Supplementary Figure 1:

Median gene expression z-score (see Methods) across age of all gene, region, cell type combinations (coronal sections) split into the nine annotated gene trajectory clusters (rows) and further divided by cell type (columns). Shaded regions correspond to interquartile range in expression. All trajectories were smoothed using interpolating B-splines.

Supplementary Figure 2:

a, Density of predicted ages computed using spatial aging clocks for different age groups in the coronal section dataset using cross-validation with groups defined by binning samples into young (less than 9 months), middle-aged (between 9 and 19 months), and old (greater than 19 months) age groups. Missing genes were imputed using SpaGE before spatial aging clock predictions were obtained. For statistical analysis, refer to Supplementary Table 12. **b**, Predicted age as a function of actual age for the sagittal section dataset using the spatial aging clocks. Heatmap colors represent density of predicted ages. Gray circles represent the median predicted age for an individual mouse. The Pearson correlation between predicted age and actual age for all cells is reported as R, and the Pearson correlation between median predicted age and actual age for all mice is reported as r.

Supplementary Figure 3:

Bar plots showing the proportion of T cells split into different subtypes defined by presence of T cell subtype marker transcripts in the MERFISH coronal section dataset.

bioRxiv preprint doi: https://doi.org/10.1101/2024.07.16.603809; this version posted July 19, 2024. The copyright holder for this preprint (which was not certified by peer review) is the author/funder, who has granted bioRxiv a license to display the preprint in perpetuity. It is made available under aCC-BY 4.0 International license.

Supplementary Figure 1

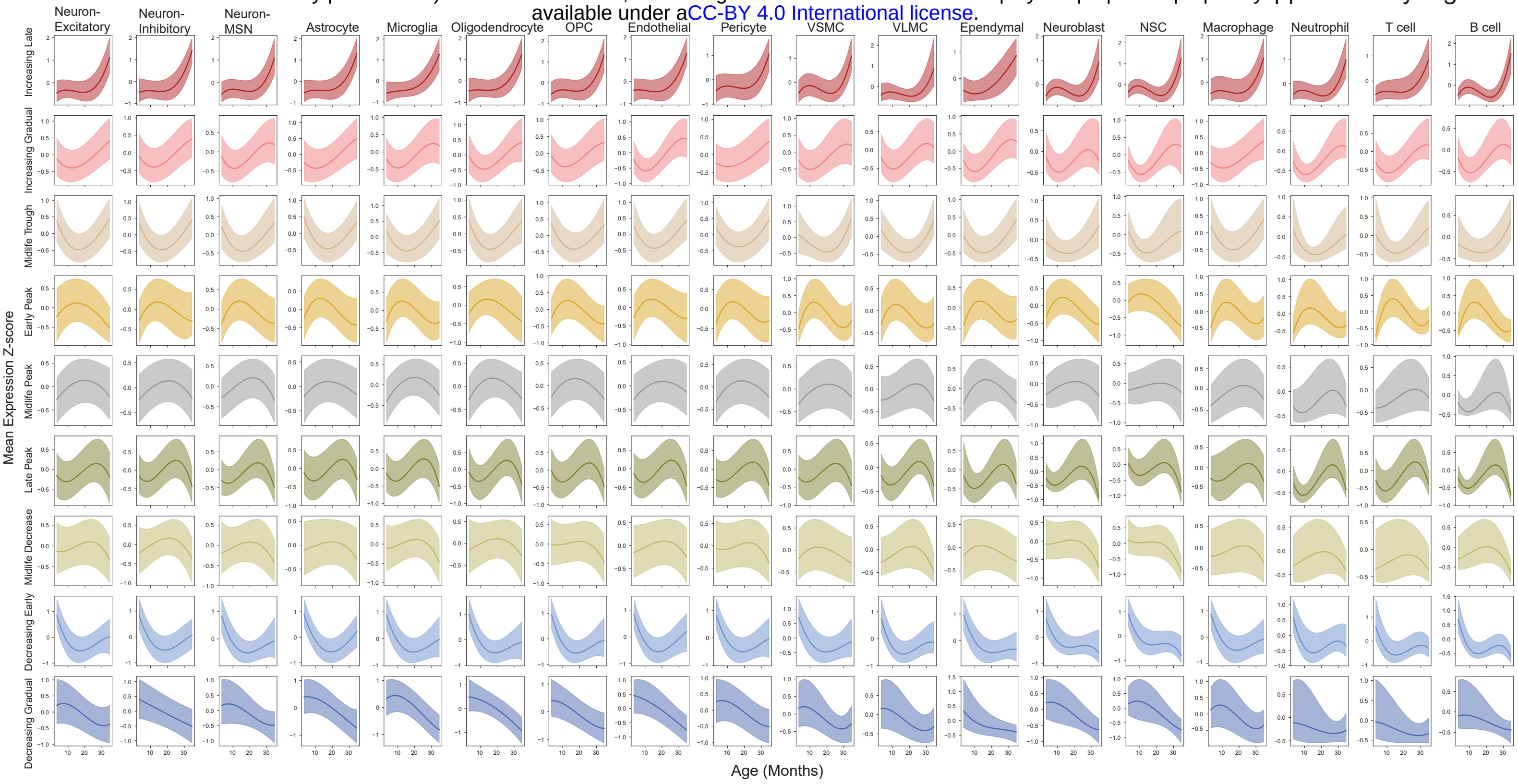

bioRxiv preprint doi: https://doi.org/10.1101/2024.07.16.603809; this version posted July 19, 2024. The copyright holder for this preprint (which was not certified by peer review) is the author/funder, who has granted bioRxiv a license to display the preprint in perpetuity. It is made available under aCC-BY 4.0 International license.

Supplementary Figure 2

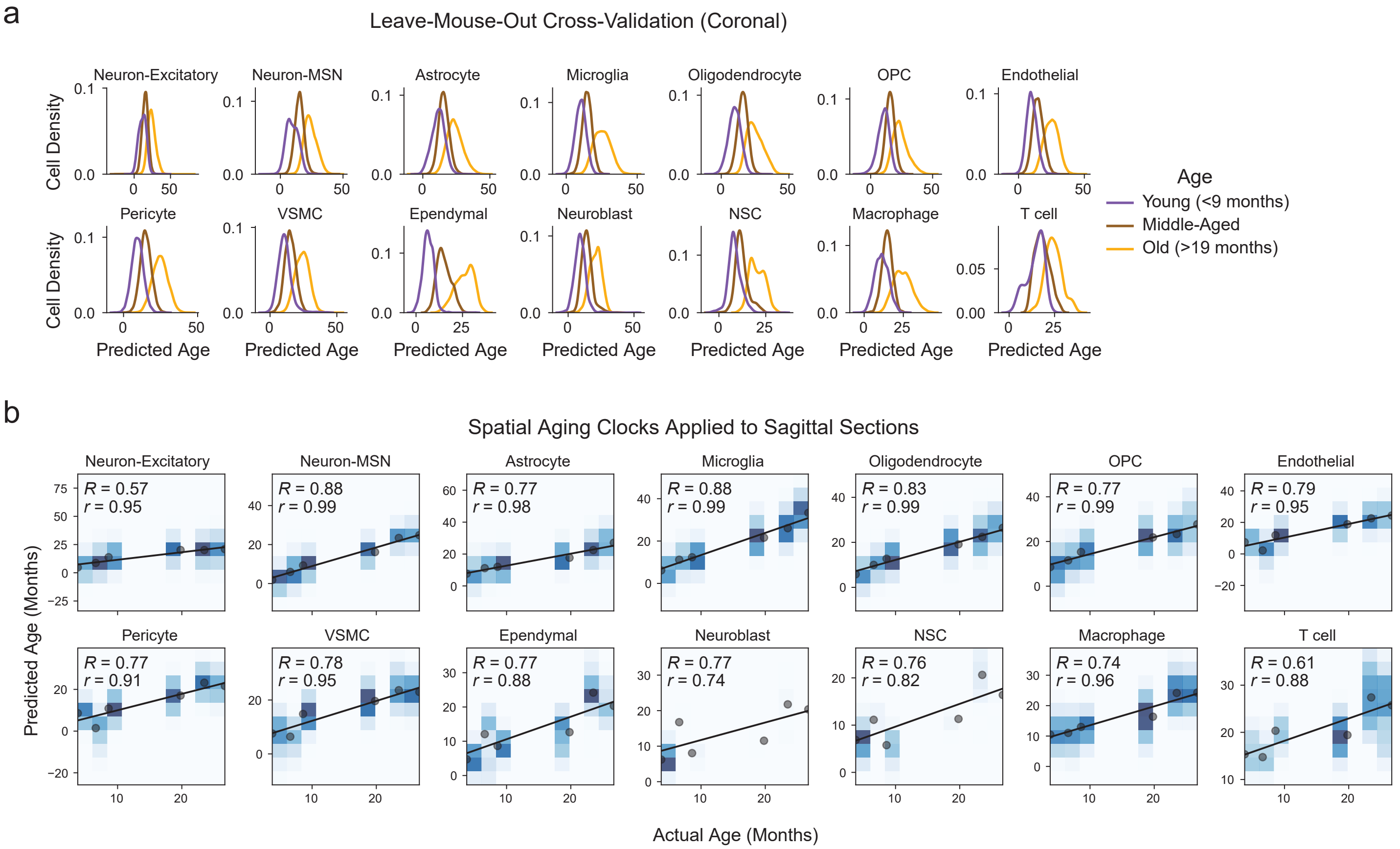

bioRxiv preprint doi: https://doi.org/10.1101/2024.07.16.603809; this version posted July 19, 2024. The copyright holder for this preprint (which was not certified by peer review) is the author/funder, who has granted bioRxiv a license to display the preprint in perpetuity. It is made available under aCC-BY 4.0 International license.

Supplementary Figure 3

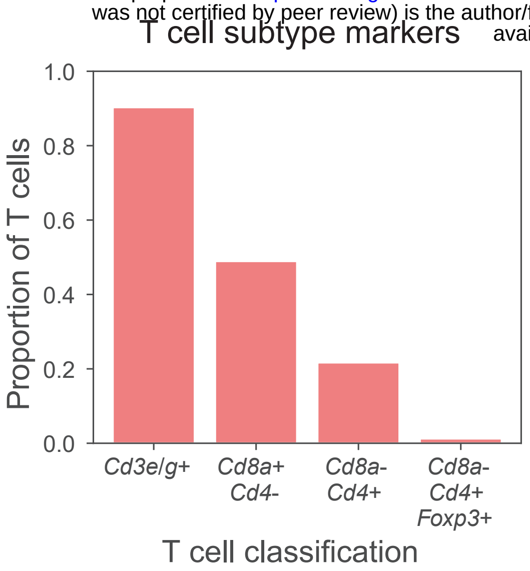

bioRxiv preprint doi: https://doi.org/10.1101/2024.07.16.603809; this version posted July 19, 2024. The copyright holder for this preprint (which was not certified by peer review) is the author/funder, who has granted bioRxiv a license to display the preprint in perpetuity. It is made available under aCC-BY 4.0 International license.

Supplementary Table 1: MERFISH gene panel containing 300 genes with information on cell type and subtype markers and additional rationale for inclusion.

Supplementary Table 2: MERFISH dataset statistics averaged across all cells in each dataset. In order, the columns correspond to the name of the dataset, the mean volume of cells, the mean transcripts detected per cell, the mean number of unique genes per cell, the mean percentage of transcripts within cell segmentations per sample, and the mean percentage of the total image area covered by cell segmentations per sample.

Supplementary Table 3: Statistics on cell filtering steps including metadata information including the dataset, mouse identifier, slide identifier, cohort/experimental condition label, and age of each sample along with the number of cells after each data preprocessing step. An additional sheet includes details and rationale for the data preprocessing steps.

Supplementary Table 4: Sample information for each mouse in the MERFISH datasets generated for the study. In order, the columns correspond to the mouse identification number, the age of the mouse in months, the slide id where ‘A’ or ‘B’ indicates the batch and the following number indicates the slide in that batch, the specific cohort/experiment associated with the mouse, the number of genes profiled, and the final number of cells identified in the sample.

Supplementary Table 5: Additional information on cell type annotation procedure for all MERFISH datasets including the final set of cell type markers used, the Leiden clustering resolution parameter used for the initial and successive rounds of clustering, and the graphical visualizations used to assist in the annotation. An additional sheet lists all cell types with markers included in the MERFISH panel and the subset of these cell types that were identified in each of our datasets.

Supplementary Table 6: Global and regional cell type proportion changes described by the Pearson correlation between cell type proportion and age across all samples for different regions, the associated P-value, and the lower and upper bounds for the 95% confidence interval for the Pearson correlation estimate. Sheets are included for the coronal section dataset and its regions and the sagittal section dataset.

Supplementary Table 7: Increasing and decreasing gene expression analysis and aging gene trajectories information for 220 genes in the MERFISH panel with low

bioRxiv preprint doi: https://doi.org/10.1101/2024.07.16.603809; this version posted July 19, 2024. The copyright holder for this preprint (which was not certified by peer review) is the author/funder, who has granted bioRxiv a license to display the preprint in perpetuity. It is made available under aCC-BY 4.0 International license.

predicted spillover rate (<5%). For each cell type, the columns ending with “PValue”, “Spearman”, “Upper95CI”, and “Lower95CI” denote *P*-value, correlation estimate, upper bound of 95% confidence interval for the correlation estimate, and lower bound of 95% confidence interval for the correlation estimate from Spearman correlation test between age and mean log-normalized expression for each gene (see Methods). For each cell type, the columns ending with region identifiers denote the categorical labels for the spatiotemporal gene expression trajectories for each gene in that region (see Methods).

Supplementary Table 8: Gene Ontology Biological Process enrichment results for all gene sets significantly enriched for genes increasing with age or decreasing with age for each cell type (Fisher’s exact test, $P < 0.05$). The table includes columns for the GO identifier, GO term, gene number statistics, *P*-value from Fisher’s exact test with the MERFISH gene panel as background, and the name of genes in each set.

Supplementary Table 9: Gene Ontology Biological Process enrichment results for all gene sets significantly enriched for genes in each of the nine spatiotemporal gene expression trajectory categories for oligodendrocytes in the CC/ACO region (Fisher’s exact test, $P < 0.05$). The table includes columns for the GO identifier, GO term, gene number statistics, *P*-value from Fisher’s exact test with the MERFISH gene panel as background, and the name of genes in each set.

Supplementary Table 10: Spatial aging clock coefficients for genes in the MERFISH dataset (rows) and for each cell type-specific aging clock (columns).

Supplementary Table 11: Gene Ontology Biological Process enrichment results for all gene sets significantly enriched for genes used in cell type-specific spatial aging clocks and separated by positive coefficient (“increasing”) or negative coefficient (“decreasing”) (Fisher’s exact test, $P < 0.05$). The table includes columns for the GO identifier, GO term, gene number statistics, *P*-value from Fisher’s exact test with the MERFISH gene panel as background, and the name of genes in each set.

Supplementary Table 12: Statistics for comparison of predicted ages between different ages and conditions. In order, the columns correspond to the figure panel, name of the dataset, the comparison evaluated in the form of ‘condition 1’ versus ‘condition 2’, the cell type, the difference in the median predicted age computed by subtracting the median predicted age in ‘condition 2’ from the median predicted age in ‘condition 1’, the statistical test used, the *P*-value obtained from the statistical test for comparing predicted ages of all cells, and the *P*-value obtained from the statistical test for

bioRxiv preprint doi: https://doi.org/10.1101/2024.07.16.603809; this version posted July 19, 2024. The copyright holder for this preprint (which was not certified by peer review) is the author/funder, who has granted bioRxiv a license to display the preprint in perpetuity. It is made available under aCC-BY 4.0 International license.

comparing median predicted ages of all mice. Additional sheets contain statistics for select comparisons when using mean imputation and when using spatial aging clocks trained on a subset of 220 genes after filtering by transcript spillover rate.

Supplementary Table 13: Gene Ontology Biological Process enrichment results for all gene sets significantly enriched for differentially expressed genes with exercise for endothelial cells (Fisher's exact test, $P < 0.05$). The enrichment results are separated for endothelial cells in the ventricles ("VEN") and for endothelial in other regions ("No VEN"). The table includes columns for the GO identifier, GO term, gene number statistics, *P*-value from Fisher's exact test with the MERFISH gene panel as background, and the name of genes in each set.

Supplementary Table 14: Results of cell proximity effect analysis for each pair of effector cell type and target cell type. In order, the columns indicate the name of effector cell type, the name of the target cell type, the t-statistic from two-sided Student's t-test on age acceleration between "Near" and "Far" cell groups, the *P*-value from two-sided Student's t-test on age acceleration between "Near" and "Far" cell groups, the cell proximity effect, and the number of pairs in the "Near" and "Far" cell groups.

Supplementary Table 15: Average cell proximity effect estimates for T cells and NSCs across multiple datasets including for all experimental conditions and for only the control conditions.

Supplementary Table 16: Total numbers of cells per cell type that are near or far from T cells and NSCs as used for imputation and in the proximity effect analysis.
